# Ballistic Performance of Ramie Fabric Reinforcing Graphene Oxide-Incorporated Epoxy Matrix Composite

**DOI:** 10.3390/polym12112711

**Published:** 2020-11-16

**Authors:** Artur Camposo Pereira, Andreza Menezes Lima, Luana Cristyne da Cruz Demosthenes, Michelle Souza Oliveira, Ulisses Oliveira Costa, Wendell Bruno Almeida Bezerra, Sergio Neves Monteiro, Ruben Jesus Sanchez Rodriguez, Janine Feitosa de Deus, Wagner Anacleto Pinheiro

**Affiliations:** 1 Military Institute of Engineering—IME, Materials Science Program, Praça General Tibúrcio 80, Urca, Rio de Janeiro 22290-270, Brazil; andrezamenezeslima@gmail.com (A.M.L.); eng.luanademosthenes@gmail.com (L.C.d.C.D.); oliveirasmichelle@gmail.com (M.S.O.); ulissesolie@gmail.com (U.O.C.); wendellbez@gmail.com (W.B.A.B.); snevesmonteiro@gmail.com (S.N.M.); anacleto@ime.eb.br (W.A.P.); 2 Laboratory of Advanced Materiais—LAMAV, State University of the North Fluminense—UENF, Avenida Alberto Lamego 2000, Campos dos Goytacazes, RJ 28013-602, Brazil; sanchez@uenf.br (R.J.S.R.); janinefd@globo.com (J.F.d.D.)

**Keywords:** ramie fabric, graphene oxide incorporation, epoxy matrix, ballistic armor, thermal analysis

## Abstract

Graphene oxide (GO) incorporation in natural fiber composites has recently defined a novel class of materials with enhanced properties for applications, including ballistic armors. In the present work, the performance of a 0.5 vol % GO-incorporated epoxy matrix composite reinforced with 30 vol % fabric made of ramie fibers was investigated by stand-alone ballistic tests against the threat of a 0.22 lead projectile. Composite characterization was also performed by Fourier-transform infrared spectroscopy, thermal analysis and X-ray diffraction. Ballistic tests disclosed an absorbed energy of 130 J, which is higher than those reported for other natural fabrics epoxy composite, 74–97 J, as well as plain Kevlar (synthetic aramid fabric), 100 J, with the same thickness. This is attributed to the improved adhesion between the ramie fabric and the composite matrix due to the GO—incorporated epoxy. The onset of thermal degradation above 300 °C indicates a relatively higher working temperature as compared to common natural fiber polymer composites. DSC peaks show a low amount of heat absorbed or release due to glass transition endothermic (113–121 °C) and volatile release exothermic (~132 °C) events. The 1030 cm^−1^ prominent FTIR band, associated with GO bands between epoxy chains and graphene oxide groups, suggested an effective distribution of GO throughout the composite matrix. As expected, XRD of the 30 vol % ramie fabric-reinforced GO-incorporated epoxy matrix composite confirmed the displacement of the (0 0 1) peak of GO by 8° due to intercalation of epoxy chains into the spacing between GO layers. By improving the adhesion to the ramie fabric and enhancing the thermal stability of the epoxy matrix, as well as by superior absorption energy from projectile penetration, the GO may contribute to the composite effective ballistic performance.

## 1. Introduction

Advanced carbon-based materials have nowadays been considered in diversified applications not only to improve existing technologies but also to benefit people’s well-being, as in the case of electromagnetic wave absorption [1,2,3]. Graphene is a typical example. A recent overview reported an exponential surge in a novel class of natural fiber polymer composites incorporated with graphene-based materials [4]. This incorporation could improve the interfacial adhesion between natural hydrophilic fibers and the hydrophobic polymer matrix [5]. Indeed, graphene has exceptional properties and great potential for innovative applications. As a monolayer of carbon atoms oriented in a hexagonal structure, graphene is a rising material with outstanding technological properties [6,7]. It offers innumerous engineering applications, either alone or combined with other materials, to form multifunctional composites [8,9,10,11]. Among the different graphene-based material stands, the graphene oxide GO [12,13]. The GO has been successful in a variety of engineering products, including electronics, conductive films, electrode materials, electromagnetic wave absorber and composites [14,15,16,17]. Owing to its amphiphilic behavior [5], GO allows easier coupling between natural fibers and polymers matrices.

Natural fiber composites are another surging class of materials [18,19,20,21,22,23,24,25,26,27,28]. These composites are being industrially applied [29,30,31,32,33] and considered as components of ballistic armor for personal protection [31,34,35,36,37]. A well-known natural fiber is that extracted from the stem of the ramie plant, *Boehmeria nivea*. Selected ramie fibers with a small diameter, ~25 µm, display tensile strength of about 1500 MPa [20]. These results motivated several research works in past decades on ramie fiber composites [38,39,40,41,42,43,44,45,46,47,48]. In particular, Marsyahyo et al. [49] presented preliminary ballistic results on bulletproof panels made of ramie woven fiber-reinforced epoxy composites indicating that they were able to resist the penetration of projectiles with an impact velocity of 380 m/s, causing only some fractures. More recently, the fabric made of ramie fibers was investigated as a reinforcing addition to epoxy composites in multilayered armor for personal ballistic protection [50,51]. In these ballistic tests, neither the ramie fabric nor the epoxy matrix was treated or modified to improve their mutual adhesion in the composite. In another recent work [52], either the curaua fiber was functionalized with GO or/and the epoxy matrix was added with 0.50% GO. It was found that composites with only GO incorporate matrix display the best performance. This motivated the present work to investigate GO incorporating only in the epoxy matrix, despite our different ramie fabric as compared with curaua fiber in the previous work [52].

A more comprehensive and deeper recent publication investigated the GO incorporation of either: (i) the natural fiber; (ii) or the epoxy matrix; (iii) or else, simultaneously, both the fiber and the matrix [53]. It was found that tensile properties of non-incorporated fiber/epoxy composite substantially increased with GO incorporation in the epoxy matrix, which was higher than the GO incorporation in the natural fiber. Moreover, GO incorporation resulted in a decrease of tensile properties in both fiber and matrices. It then became clear that the incorporation of GO only in the polymer matrix may be the most effective way to improve the composite mechanical behavior [53]. Based on the aforementioned finding, the present work investigates for the first time the ballistic performance of non-functionalized ramie fabric-reinforced GO-incorporated epoxy matrix composites, motivated by previous ballistic [52] and mechanical results [53] obtained in curaua fiber composites.

## 2. Materials and Methods

### 2.1. Materials

**Figure 1:**
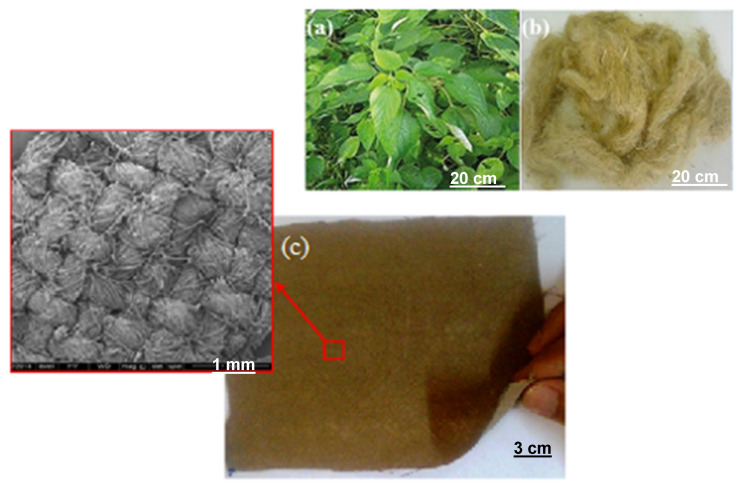
The *Boehmeria nivea* plant (**a**), a bunch of nonwoven ramie fibers (**b**) and the ramie fabric (**c**). Reproduced with permission from [38,51].

The polymer used as a matrix was a commercially available epoxy resin, diglycidyl ether bisphenol-A (DGEBA), hardened with triethylene tetramine (TETA), using the stoichiometric ratio of 13 parts of hardener per 100 parts of resin. Both resin and hardener were manufactured by Dow Chemical and supply by Epoxy Fiber (Rio de Janeiro, Brazil). The ramie fabric was purchased from Rose Natural (China). The areal density of the ramie fabric was reported as 245 g/m^2^ [51]. Figure 1 illustrates (a) the *Boehmeria nivea* plant, (b) a bunch of nonwoven ramie fibers and (c) the ramie fabric. The ramie fabrics were cut to 150 mm in length and placed in an oven at 80 °C for 24 h until the weight of the fiber remained stable, without moisture.

The GO used in this work was produced by the modified Hummers method, according to the procedure employed by Rourke et al. [16]. The GO with 0.56 mg/mL of concentration went through a drying process in a vacuum pump to eliminate the water present in the material. Then, the GO was mixed in isopropyl alcohol in a proportion of 2:1. This solution was added to the epoxy resin and placed in an oven at 70 °C for 24 h to eliminate the alcohol. Chemical analyses were performed to assure that the solution contained no alcohol as a routine procedure [52,53]. Finally, the epoxy resin with 0.5 vol % of GO received the hardener for making the composite.

### 2.2. Composite Processing

The composite plate was manufactured using a metal mold with dimensions of 150 × 120 × 12 mm. The amount of ramie fabric corresponding to 30 vol % was laid inside the mold, and the still fluid DGEBA/TETA was poured into the mold, which was then closed and subject to pressure in a SKAY (São Paulo, Brazil) hydraulic press with a load of 5 tons for 24 h.

### 2.3. Ballistic Test

**Figure 2:**
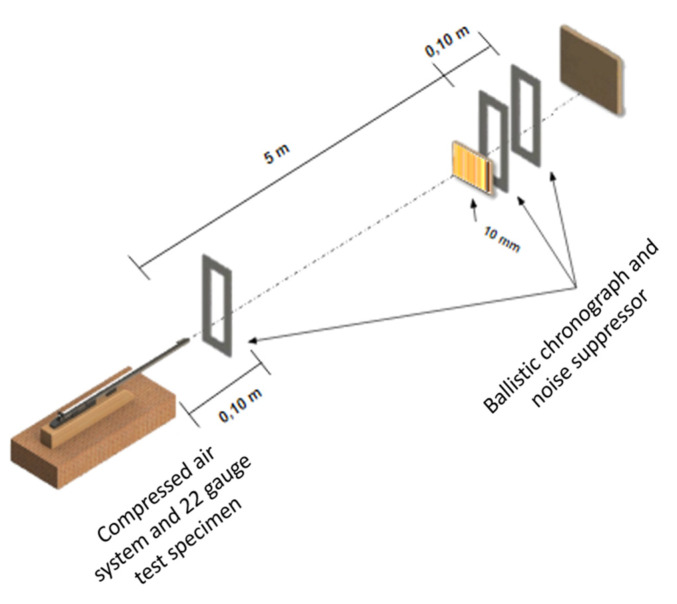
System used in the ballistic test.

A ballistic test was performed to evaluate the kinetic energy absorption capacity by the investigated composite plate. The original composite plate was cut to dimensions 12 × 7 × 1.2 cm for the test. A Gunpower SSS sniper rifle (Ashford, UK) with a weapon standard noise suppressor was used. The projectile was a 0.22-gauge rifle bullet with a mass of 3.3 g. The air rifle was positioned 5 m away from the target, consisting of a plate attached by a vise and aligned perpendicularly to the rifle. One ballistic chronograph was placed 10 cm before the target, and the other was placed 10 cm behind the target. The system used for this test is shown in Figure 2.

To determine the absorption energy, an Air Chrony ballistic chronograph model MK3 (Move Mesto, Czech Republic), with a precision of 0.15 m/s, was used to measure the velocity of the impact, and a ProChrono ballistic chronograph model Pal (Rockford, IL, USA), with a precision of 0.31 m/s, was used to measure the residual velocity.

The energy absorbed by the target was calculated as:

(1) $$E_{abs}=\frac{m_{p}\left(v_{i}^{2}-v_{r}^{2}\right)}{2}-E_{0}$$

where *m_p_* is the mass of the projectile, *v_i_* is the impact speed, *v_r_* residual speed and *E*_0_ is the energy dissipated by the projectile only by air-flying without a target.

### 2.4. X-ray Diffraction

For X-ray diffraction analysis, both a fraction of approximately 100 mg in powder form and a solid piece were removed from the composite plate. The parameters used in this analysis, conducted in an X`Pert Pro Panalytical diffractometer (Malvern, UK), were operated with a voltage of 40 kV and a current of 40 mA in the range of 1 h; 2θ angles from 5 to 80° with a step of 0.05° and radiation CuKα, at room temperature.

### 2.5. Fourier-Transform Infrared Spectroscopy (FTIR)

Fourier-transform infrared spectroscopy analyses were performed in a Frontier FT-IR/FIR equipment, ATR accessory (Waltham, MA, USA), 4 cm^−1^ resolution, 60 scans, in the wavenumber range between 4000 and 600 cm^−1^.

### 2.6. Thermal Analysis

The thermogravimetry (TGA) analysis was performed on a TA Instruments Systems TGA Q500 (New Castle, DE, USA) equipment. The analysis took place in a nitrogen atmosphere, at a heating rate of 10 °C/min and in a temperature range of 25 to 800 °C. The DSC was analyzed in a TA Instruments Systems Q1000 (New Castle, DE, USA) equipment, using a heating rate of 10 °C/min and a temperature range between 25 and 250 °C.

### 2.7. Scanning Electron Microscope (SEM)

Microscopic analyses of the composite fracture were performed by scanning electron microscopy (SEM) in a model Quanta FEG 250 Fei microscope (Hillsboro, OR, USA), operating with secondary electrons using acceleration voltages between 5 and 15 kV.

## 3. Results and Discussion

### 3.1. Ballistic Test

**Figure 3:**
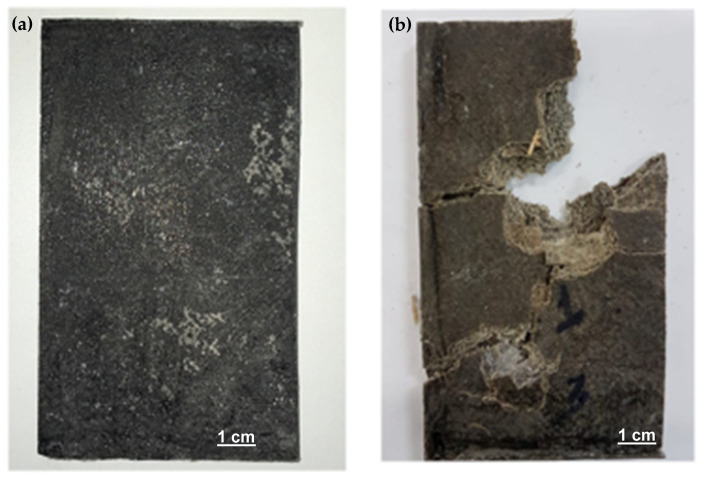
Image of the sample before (**a**) and after ballistic impact (**b**).

The ballistic test showed that the composite target started suffering fractures since the first shooting. Figure 3 illustrates the conditions that the composite plate suffered after all shootings.

Table 1 presents the average values of the composite mass (*M_c_*), projectile mass (*m_p_*), average impact speed (*v_i_*), average residual speed (*v_r_*) and the absorption energy (*E_abs_*) of each composition. Table 2 shows the values of different composites with their respective absorption energy values. With ballistic tests using the same configuration and target thickness, composites with coconut/epoxy [54], sisal fabric/epoxy [55], a hybrid composite of Kevlar/coconut/epoxy [54] and composite with *Cyperus malaccensis*/epoxy [36] showed lower values than the present composite with GO-incorporated epoxy matrix. By comparing the absorption energy of other epoxy composites in Table 2, the addition of GO in the epoxy matrix reveals a superior ballistic performance of the present 30 vol % ramie fabric composite.

**Figure 4:**
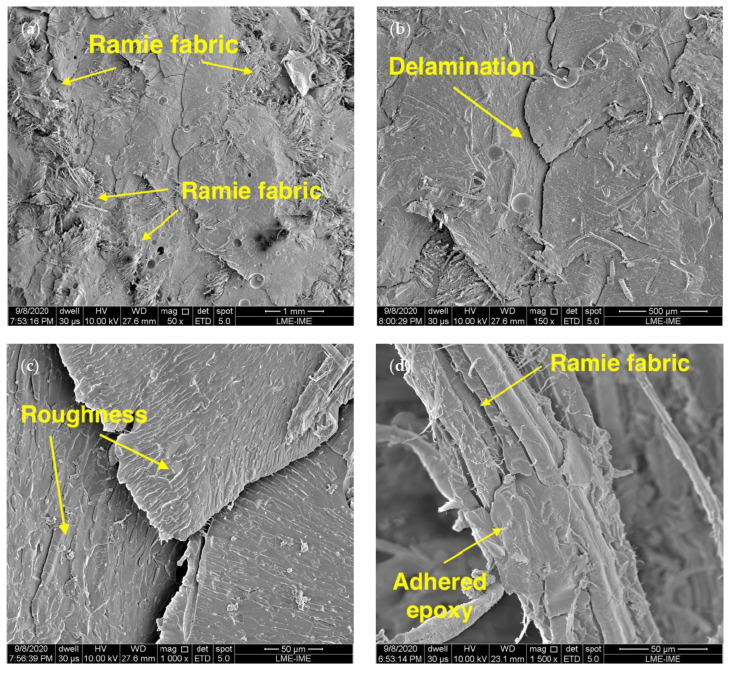
SEM of the fracture surface of the ballistic test with an increase of (**a**) 50×, (**b**) 150×, (**c**) 1000× and (**d**) 1500×.

Figure 4 shows SEM micrographs of the composite fracture. The ballistic test in Figure 4a depicts total fiber fracture without the pullout effect. However, even though the GO was applied to the epoxy resin, there was a good fiber adhesion with the matrix [5,56]. Figure 4d shows the epoxy matrix encrusted in the ramie fiber, revealing an improvement in adhesion.

In Figure 4b,c, the GO epoxy resin surface is presented in more detail. Unlike a fracture surface of a pure epoxy resin, which is smooth, the surface of the composite showed a rough fracture. This roughness may cause stress concentration and alter mechanical performance [57,58]. It could also interfere in the crack propagation and increase the absorbed ballistic impact energy.

### 3.2. Thermogravimetric Analysis (TGA)

**Figure 5:**
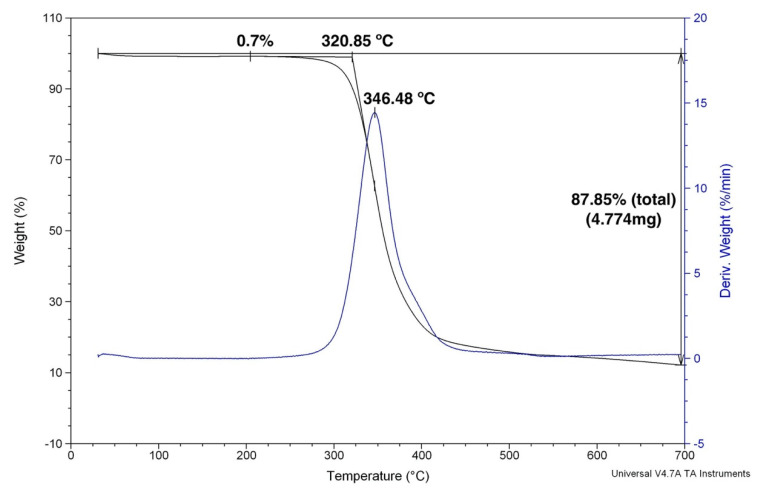
TGA curves for the plain epoxy used as a composite matrix.

**Figure 6:**
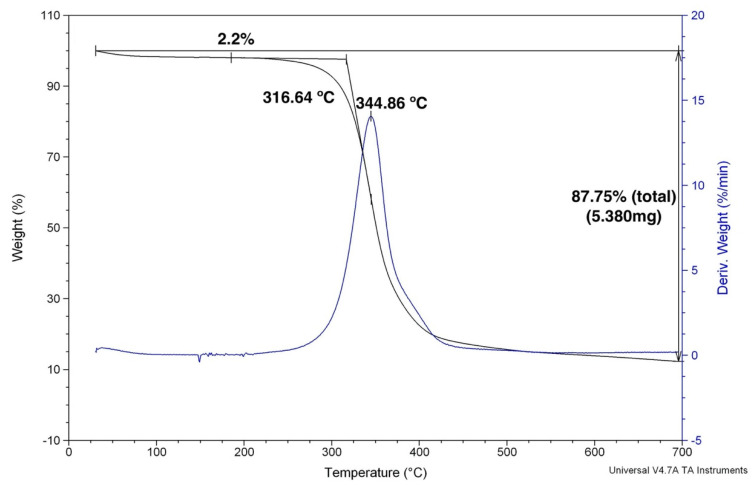
TGA curves for the graphene oxide (GO)-free epoxy composite reinforced with 30 vol % of ramie fabric.

Figure 5 shows the TG and first derivate (DTG) curve for the plain epoxy. As expected, practically no mass loss due to moisture release occurred in the hydrophobic epoxy up to 250 °C. Macromolecular chain decomposition was accentuated above 320.9 °C, reaching a maximum rate of degradation at 346.5 °C and a marked 87.75% loss of mass at 700 °C. The incorporation of 30 vol % ramie fabric into GO-free epoxy matrix composite was associated with the TG/DTG curves in Figure 6. In this figure, an initial mass loss of 2.2% was attributed to moisture release from the hydrophilic ramie fibers in the fabric. The onset of the composite degradation began at a lower temperature, 316.6 °C, than that of the plain epoxy in Figure 5. This may be assigned to the ramie fiber cellulose, hemicellulose and lignin beginning of thermal decomposition. As for the maximum rate of degradation at 344.9 °C, it was practically equal to that in Figure 5 and probably due to the same thermal degradation mechanism of plain epoxy.

**Figure 7:**
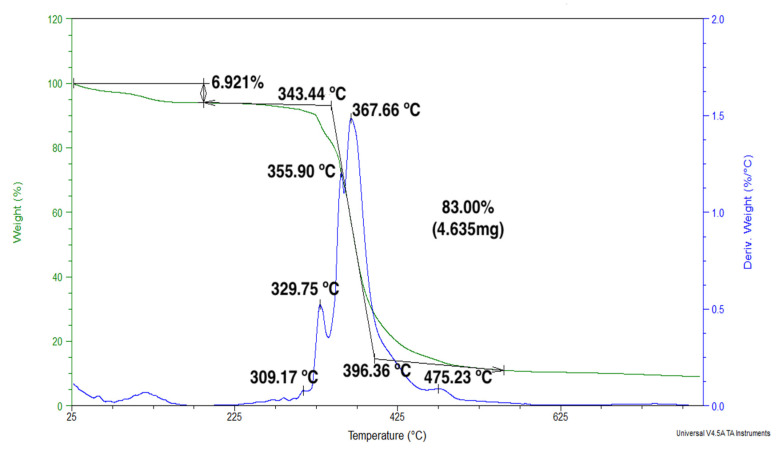
TGA and first derivate (DTG) curves of 30 vol % ramie fabric GO-incorporated epoxy composite.

In Figure 7, it is possible to observe an initial mass loss of 3.7%, which is associated with a small change of level in the TG curve. This loss is attributed to the evaporation of the moisture present in natural fibers [21,22,23,24,25,26,27,28]. After this stage, it is possible to observe a more marked loss of mass of 82.87% in total, starting at a temperature of approximately 343 °C. According to previous studies in natural fibers [59,60], this variation was related to the degradation of the components of the fibers, namely: hemicellulose, cellulose and lignin. It is noteworthy in Figure 7 that both the onset, 343.4 °C, and the maximum rate of degradation, 367.7 °C, were higher than those for both the plain epoxy in Figure 5 and the GO-free ramie fabric epoxy composite in Figure 6. This is evidence that the incorporation of GO into the epoxy matrix provides enhanced thermal resistance to the composite. In the DTG curve of ramie fabric and GO-incorporated epoxy composite in Figure 7, it is possible to individually observe the different rates of degradation present during the maximum rate of loss of mass observed. The maximum rate of degradation peaks present at 309.2 and 475.2 °C may be associated with the decomposition of hemicellulose and lignin, respectively [59]. In the intermediate temperature values, the observed peaks may be attributed to the degradation of the fiber cellulose and the epoxy resin reinforced with GO [41,60], since it was observed that the addition of GO does not alter the decomposition mechanism in the epoxy matrix [4,57]. Regarding the TGA results in Figure 7, one may consider the working temperature for this novel ramie fabric-reinforced GO-incorporated composite to be 300 °C. This indicates a higher thermal resistance than fiber composites usually applied in ballistic armors for personal protection.

**Figure 8:**
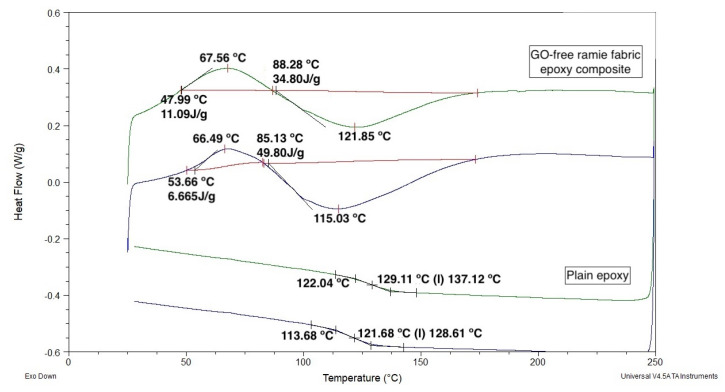
DSC curves for one heating and cooling cycle of plain epoxy (lower side) and GO-free epoxy matrix composite reinforced with 30 vol % ramie fabric (upper side).

Figure 8 shows the DSC obtained for both heating and cooling runs of plain epoxy and GO-free ramie fabric epoxy composites. The plain epoxy endothermic peaks (122–129 °C) may be attributed to the interval of glass transition temperature (*T_g_*). As for the composite, the endothermic peak at ~67 °C is associated with the highest rate of moisture release. While the endothermic peaks (115–129 °C) correspond to the epoxy *T_g_*, indicating only a slight effect of the ramie fabric on the organization of the molecular matrix chains.

**Figure 9:**
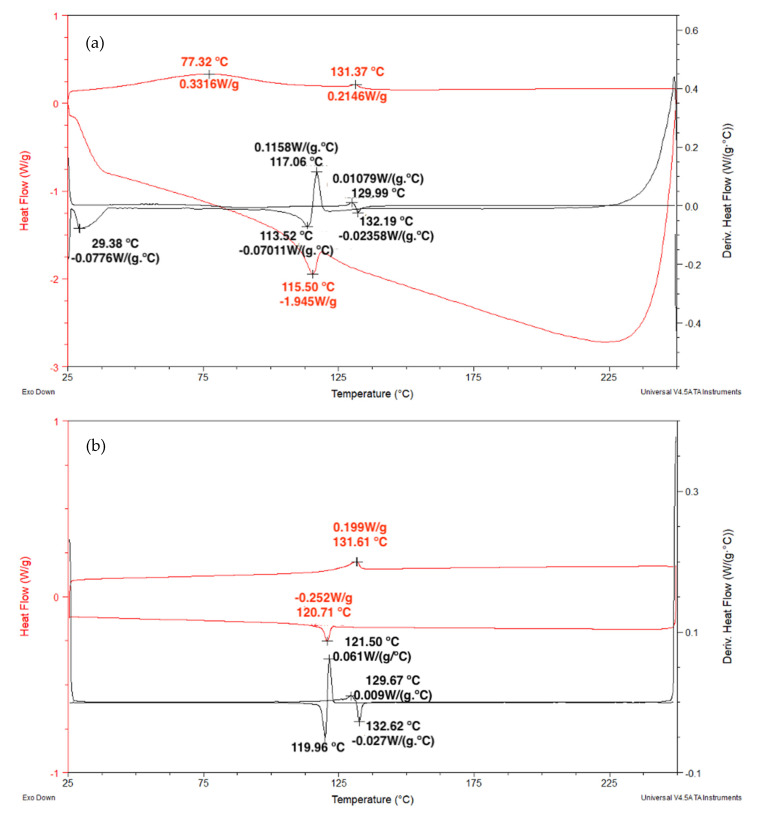
DSC curves obtained for the epoxy resin composite reinforced with GO and ramie fabric: first (**a**) and second (**b**) heating and cooling cycles.

Figure 9a,b shows the DSC curves obtained after two heating and cooling cycles for the samples of the ramie fabric GO-incorporated epoxy composite. It is possible to observe in Figure 9a the presence of two small exothermic peaks. The first of these peaks was found at 77.3 °C. This peak was assigned to the evaporation of moisture in the sample [61]. The other exothermic peak was observed at 131 °C on the DSC curves. This peak may be associated with the beginning of the decomposition of the ramie fibers in the fabric, promoting the release of volatiles [59]. In addition, endothermic peaks were also observed during the first cycle performed. These peaks, associated with low absorbed heat, were observed at 115.5 and 113.5 °C, respectively, and correspond to the epoxy *T_g_*. The second heating and cooling cycle in Figure 9b obviously showed no more water release but still displayed residual release of volatiles associated with a small exothermic peak at 131.6 °C. As for the endothermic peaks in this figure, they were slightly displaced to higher temperatures, 120.0 to 120.7 °C, as compared to the first cycle in Figure 9a. These values were still within the *T_g_* interval for GO-free ramie fabric epoxy composite in Figure 8. As such, the 0.5 vol % of GO-incorporated caused no apparent change in the DSC behavior, particularly in the *T_g_* of the composite.

### 3.3. Fourier-Transform Infrared Spectroscopy (FTIR)

**Figure 10:**
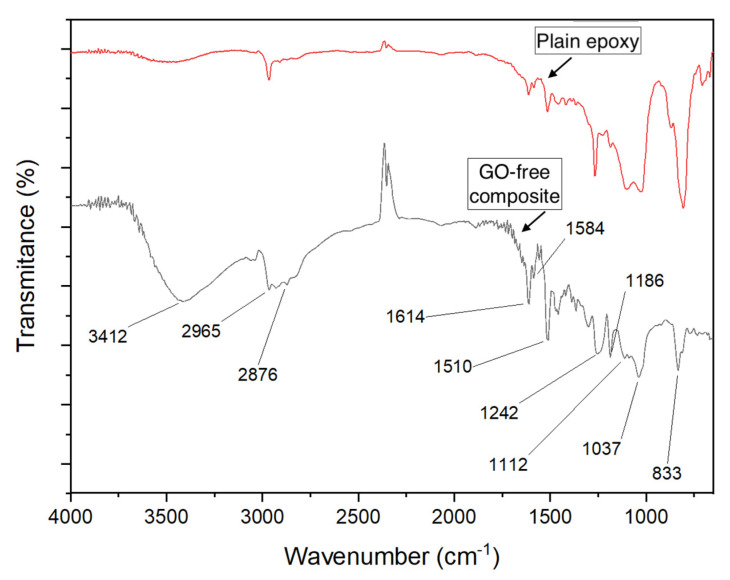
FTIR spectra of plain epoxy and GO-free 30 vol % ramie fabric epoxy composite.

Figure 10 shows the FTIR spectra of the plain epoxy and the GO-free ramie fabric epoxy composite. The main bands in this figure are found in both spectra but with different intensities due to the epoxy interaction with the ramie fibers. The extended band around 3412 cm^−1^ is attributed to the stretching of the O–H bond existing in both epoxy and ramie fabric. Similar band was much more accentuated in the ramie fabric due to H_2_O molecules present in fiber cellulose and hemicellulose [41]. The set of bands between 2965 and 2876 cm^−1^ are basically assigned to CH_2_ vibration in cellulose and hemicellulose of the ramie fiber [41]. Bands at 1614, 1584 and 1510 cm^−1^ are related to the C=O in the benzene ring or the C–C elongation bond of the aromatic ring in the epoxy [62,63]. As for the bands at 1242 and 1186 cm^−1^, they refer to the stretching of C–O–C bands of epoxy, but mostly in the phenolic groups present in the ramie fiber constituents [41]. On the other hand, bands at 1112 and 1037 cm^−1^ have been assigned to C–O stretching vibration in the epoxy chain [52,64,65]. Finally, the band at 833 cm^−1^ is associated with stretching C–O–C of the oxirane group [66].

**Figure 11:**
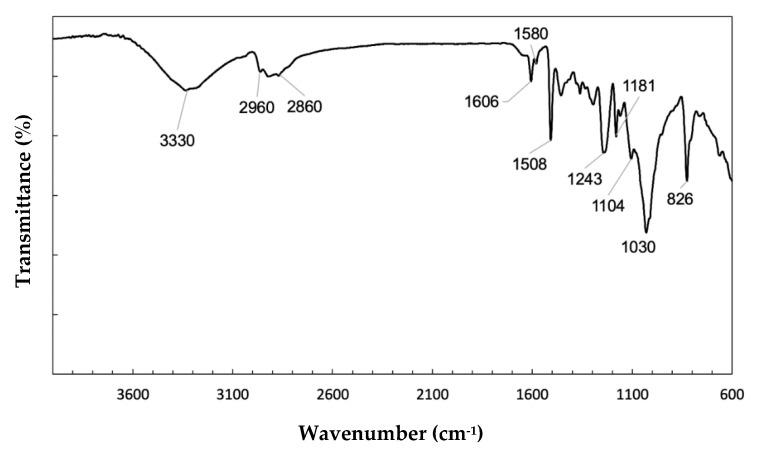
FTIR spectrum obtained for the 30 vol % ramie fabric-reinforced GO-incorporated epoxy composite.

Figure 11 depicts the FTIR spectrum of the 30 vol % ramie fabric-reinforced GO-incorporated epoxy composite. The bands in this spectrum are practically the same for the GO-free composite in Figure 10. A striking difference is the greater intensities of the bands at 1243 cm^−1^, 1104 cm^−1^ and 1030 cm^−1^ for the GO-incorporated epoxy composite in Figure 11 as compared with the corresponding ones for the GO-free composite in Figure 10. These bands involve the vibration of O-containing groups in which their intensities were obviously enhanced by the GO [64]. This may be interpreted as an effective interaction of the GO with the epoxy and probably a good distribution of GO in the composite matrix, which contributes not only to enhance mechanical properties but also to improve the ballistic performance of the GO-incorporated composite [4].

**Figure 12:**
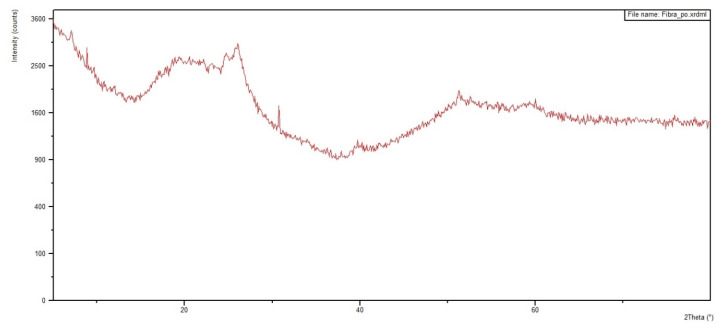
Diffractogram of the 30 vol % ramie fabric-reinforced GO-incorporated epoxy composite in the physical state of powder.

**Figure 13:**
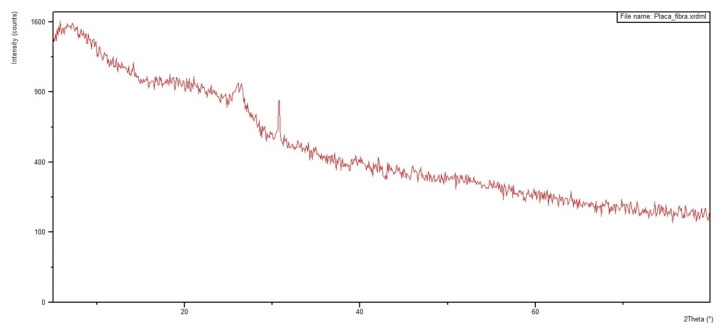
Diffractogram of the 30 vol % ramie fabric-reinforced GO-incorporated epoxy composite in the physical state of a solid plate.

**Figure 14:**
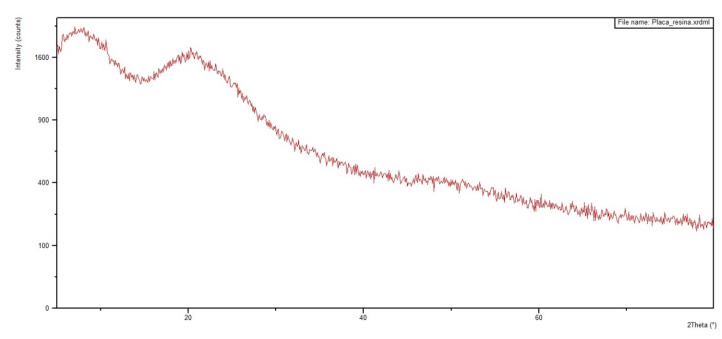
Diffractogram of plain epoxy resin.

**Figure 15:**
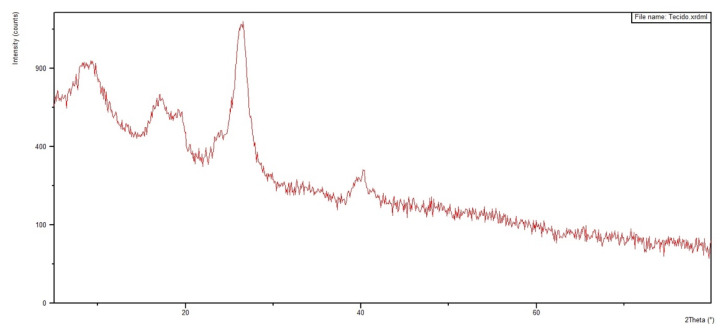
Diffractogram of the plain ramie fabric.

Figure 12 and Figure 13 show the diffractograms obtained for the 30 vol % ramie fabric GO-incorporated epoxy matrix composite with two physical conditions: in powder form and in a solid plate. Analyses were performed on both configurations to obtain clearer information. In addition, the diffractograms of pure epoxy resin and ramie fabric are shown in Figure 14 and Figure 15. The epoxy resin, with its peak at approximately 2θ = 25°, displaces the reflection peak of the GO (001) by 2θ = 8° because of the intercalation of the epoxy resin chains in the spacing between the GO layers [67]. The presence of peaks referring to the crystalline planes, characteristic of lignocellulosic materials, approximately 2θ = 28°, suffered great dispersion in the presence of epoxy resin. These behaviors are common in polymeric composite materials, and XRD analysis is not necessarily a better tool for determining dispersion homogeneity. The use of the high magnification electron microscope can help with these data [12,64], which is being conducted in our ongoing research work.

The XRD results in Figure 12, Figure 13, Figure 14 and Figure 15 are not conclusive regarding the advantage of using the GO-incorporated epoxy matrix. However, they do not reveal any shortcomings for the possible use of this novel composite in multilayered ballistic armor [31,34,35,36,37].

## 4. Summary and Conclusions

- In an unprecedented way, the ballistic performance of the composite with 0.5 vol % of GO in the epoxy matrix reinforced with 30 vol % of ramie fabric was evaluated and the composite characterized by thermal, FTIR and XRD analyses.
- Ballistic tests showed an increase in absorption energy. It was highlighted that the composite was tougher in the presence of GO in the epoxy matrix and increased the ballistic energy absorbed when compared to other composites.
- The images obtained by SEM revealed improvement in the fiber/matrix interface with the presence of GO. The pullout effect of the fibers in the matrix was not observed. In addition, the epoxy fracture surface showed roughness and separation of platelets that contributed to enhancing the mechanical properties.
- Thermal analyses by TG and DTG curves showed a degradation that starts at 343 °C with an 82% weight loss. In addition, the degradation peaks between 309 and 475 °C did not change in the presence of GO in the decomposition mechanism of the epoxy matrix. A working temperature of 300 °C may be assigned to this novel composite indicating higher thermal resistance than fiber composite usually applied in ballistic armors. Furthermore, DSC analysis demonstrated exothermic peaks at around 77 and 131 °C; and endothermic peaks during the cooling cycle at around 115 and 120 °C associated with the composite glass transition temperature.
- The FTIR pointed out the existence of OH to CH bands of the ramie fabric and the CO bands from the epoxy and GO. The presence of the same bands in the different samples in similar proportions of transmitted intensity may indicate that there was a good distribution of GO in epoxy resin composites reinforced with ramie fabric.
- The composite XRD diffractogram proved the tendency of the epoxy resin to reflect the GO characteristic peaks. The presence of the ramie fabric peaks was detected at approximately 28°.
- As compared with numerous natural fiber and fabric-reinforced polymer composites, the novel ramie fabric-reinforced GO-incorporated epoxy composite is a promising material for the second layer in a ceramic front multilayered ballistic armor for personal protection.

## Figures and Tables

**Table 1:** Absorption energy test.

Specimen	*m_p_* (g)	*v_i_* (m/s)	*v_r_* (m/s)	*E_abs_* (J)
No target (*E*_0_)	3.36 ± 0.05	285.83 ± 5.28	283.77 ± 2.38	2.69 ± 1.71
Composite	3.36 ± 0.03	283.23 ± 6.63	42.67 ± 32.10	130.34 ± 9.51

**Table 2:** Energy absorption in ballistic tests of different composite materials.

Composite	*E_abs_* (J)	Reference
Ramie fabric/0.5% GO epoxy	130.34	Present Work
Coconut fabric/epoxy	78.58	[54]
Sisal fabric/epoxy	96.84	[55]
Kevlar/coconut/epoxy	79.53	[54]
*Cyperus malaccensis*/epoxy	74.0	[36]
Kevlar	99.74	[54]
